# Experience of Healthcare Access in Australia during the First Year of the COVID-19 Pandemic

**DOI:** 10.3390/ijerph182010687

**Published:** 2021-10-12

**Authors:** Tegan Podubinski, Louise Townsin, Sandra C. Thompson, Anna Tynan, Geoff Argus

**Affiliations:** 1 Department of Rural Health, The University of Melbourne, Wangaratta, VIC 3677, Australia; 2 Research Office, Torrens University Australia, Adelaide, SA 5000, Australia; louise.townsin@torrens.edu.au; 3 Western Australian Centre for Rural Health, University of Western Australia, Geraldton, WA 6531, Australia; sandra.thompson@uwa.edu.au; 4 Darling Downs Health, Toowoomba, QLD 4350, Australia; anna.tynan@health.qld.gov.au; 5 Southern Queensland Rural Health, The University of Queensland, Toowoomba, QLD 4350, Australia; g.argus@uq.edu.au

**Keywords:** healthcare access, healthcare utilization, COVID-19, mental health, telehealth

## Abstract

Changes in health-seeking behaviours and challenges in accessing care have been reported during the COVID-19 pandemic. This qualitative study examines Australian experiences related to healthcare access during the early months of the pandemic. The study aimed to identify key areas of concern as well as opportunities for services to prevent, manage and treat health concerns when normal access was disrupted. Fifty-nine semi-structured interviews were analysed. Participants were interviewed between August and December in 2020 over telephone or Zoom and were located across Australia. Rapid identification of themes with an audio recordings technique was used to generate themes from the data. Participants described a variety of influences on their health-seeking behaviours, resulting in decisions to delay care or being unable to reach care. Many individuals accessed health services via telehealth and offered a range of perceptions and views on its effectiveness and appropriateness. The findings illustrate that maintenance of health and access to healthcare and psychosocial support were compromised for some individuals, leading to negative impacts on both mental and physical health. This highlights the need to provide mechanisms to facilitate a person’s ability to access care in a timely manner during a pandemic.

## 1. Introduction

In response to COVID-19, governments around the world have introduced a range of measures to mitigate and contain transmission of the disease including social distancing, quarantine and lockdown orders [1]. The COVID-19 pandemic has also caused widespread disruptions to usual healthcare events, resulting in decreased admissions, imaging evaluations and attendance at emergency departments [2,3,4,5,6,7]. Changes in healthcare utilization due to the pandemic are evident for both the provision of healthcare services (supply) and consumer ability to access those services (demand) [8]. Previous pandemic conditions, such as the Ebola and SARS outbreaks, also resulted in a decline in overall healthcare utilization [9,10].

While health is not reliant upon the provision of health services, modern healthcare has a role in both the primary and secondary prevention of disease and in curing some conditions or reducing disease consequences that affect morbidity, mortality and quality of life. Hence, access to care is important and needs to be broadly understood [11,12]. Outside of pandemics, access to healthcare services is a complex issue but can be defined as the “opportunity or ease with which consumers or communities are able to use appropriate services in proportion to their needs” [12] (p. 1). The dimensions and determinants of access have been the subject of many discussions, with both demand and supply factors needing to be integrated and considered; for example, having a service available versus the consumer being able to engage with the service [12]. Prior to the pandemic and within Australia, gaps in equitable access to healthcare already existed, particularly for vulnerable populations such as rural and remote and Indigenous communities [13,14]. Thus, it is important to consider the additional disruption COVID-19 has had on access to healthcare unrelated to COVID-19 illness.

In Australia, the burden of COVID-19 cases has not been as high as in many other countries around the world. By mid-2020, except for the state of Victoria, locally acquired cases were the minority of infections, with the majority acquired from people returning from overseas and diagnosed in quarantine settings [15]. Still, reduced healthcare utilization by Australians has been reported across the tertiary and primary health settings, particularly during the first year of the pandemic [16,17]. When people fail to access the care they need, the impact can be seen in an increase in both direct mortality (from the disease itself) and indirect mortality (from preventable and treatable conditions) [18,19,20,21]. Such consequences can either be exacerbated or mitigated through individual patient and health service responses [22]. For example, during the COVID-19 pandemic, healthcare workers adopted a variety of strategies, including telehealth and home delivery for medicines, to promote continuation of care and reduce the risk of exposing patients to infections. The World Health Organization provides an operational guide to maintain essential health services in the COVID-19 context but notes that successful implementation of any strategies will require the active engagement of communities with community voices essential to inform the adaptation of services so that they are responsive to local needs [18].

Elderly people and individuals with pre-existing chronic medical conditions are at increased risk of morbidity and mortality due to COVID-19 [23,24]. However, excess deaths, including out-of-hospital deaths, from non-COVID conditions such as cancer, diabetes, dementia, hypertension and heart disease have occurred during the pandemic [25,26,27,28]. Thus, COVID-19 has particular implications for the ability of vulnerable populations to access healthcare, as they will need to balance specific care needs against a heightened risk of contracting the disease. Thus, strategies to promote continuation of care will need to be developed to cope with the specific needs of vulnerable populations. This balance is likely to become more precarious as the pandemic continues and local COVID-19 transmission scenarios and health system capacity evolve [18].

The economic, societal and clinical consequences of changes in healthcare utilization are critical topics of inquiry, involving multiple, complex factors. In this respect, research into the experiences and perceptions of access to healthcare during COVID-19 is timely and important even in low-burden settings. This paper forms part of a larger program of research investigating how Australians who have identified as having chronic diseases experienced the restrictions related to COVID-19. While multiple insights were generated from this broader investigation, in this paper, we aim to describe Australian healthcare utilization experiences during the first eight months of the COVID-19 pandemic. This includes barriers to accessing healthcare and coping mechanisms used by Australians to ensure continuation of care. The findings will help healthcare services to continue to work towards a more accessible health system and could be helpful in discussions of future health service policies related to pandemics.

## 2. Materials and Methods

### 2.1. Methodological Approach

The methodological approach for this paper was aligned with Neal et al. [29]. This body of work describes the management of large-scale interviewing and coding in dynamic contexts. They outline a five-step procedure for the rapid identification of themes from audio recordings (RITA), from identifying research foci, through deductive and iterative coding, to the assessment of coding reliability between investigators. The aim of RITA is to facilitate rapid coding as a first layer of analysis, whilst simultaneously supporting the researcher to re-visit the data repeatedly for detailed notes and analysis. It can be argued that this repeat listening and extensive note-taking both during and after the interview provides for deeper analysis. The methodological approach to this study, along with the challenges and strengths of this approach have been reported in detail [29,30].

### 2.2. The Research Team

This large-scale project involved 20 collaborators from the Australian Rural Health Education Network (ARHEN). All collaborators completed interviews. The entire team lived through the pandemic in Australia, and each individual was subject to the relevant restrictions implemented in their state. To manage different investigator experiences, the team engaged in reflexive practice by regular check-ins and discussions, thereby creating a joint understanding of the team’s influence on the process of data collection and analysis. The entire team received training in the use of the RITA methodology prior to the commencement of interviewing.

### 2.3. Participant Sampling and Recruitment

Participants for the qualitative interviews were selected from a larger pool of respondents who had completed an online, nationally distributed survey investigating concerns around transmission and compliance with isolation, hygiene and social distancing measures implemented by the federal and state governments at the time of national lockdown. Only participants who had expressed a willingness to being interviewed were considered. The investigators employed purposive and convenience sampling to determine a pool of participants for interviews. It was decided to sample only survey respondents who had reported living with a chronic illness at the time of completing the survey; this group had been explicitly identified in public health and political messaging as being particularly vulnerable to the impacts of COVID-19, yet, individual responses to a question about perception of vulnerability on the survey generated inconsistent answers.

Invitations to a 30-min interview and participant information forms were emailed in August 2020 to the 172 potential participants. Emails were sent by the project coordinator, who also provided each investigator with the contact details of a negotiated number of potential participants. To minimize investigator bias, no investigators were allocated participants from their own region.

### 2.4. Interviewing

Ninety semi-structured interviews were conducted between August and December 2020 via phone or video conference. No face-to-face interviews were offered or conducted due to the evolving nature of COVID-19 restrictions. All interviews were audio recorded with the participant’s permission; verbal consent to recording was captured on the recording before commencing each interview. The collaborative team had prepared a series of questions that were used by all investigators to prompt discussion. These questions were designed to reflect the themes from the initial survey, as well as participant experience of the pandemic and associated restrictions and impact on daily life. The interview schedule is shown in Table 1.

The interview questions were provided to each investigator as part of a briefing document that provided information and support for all interviewers. The briefing document was circulated via email and discussed via a 1:1 video call between each investigator and the project coordinator.

### 2.5. Organisation of Data

**Figure 1:**
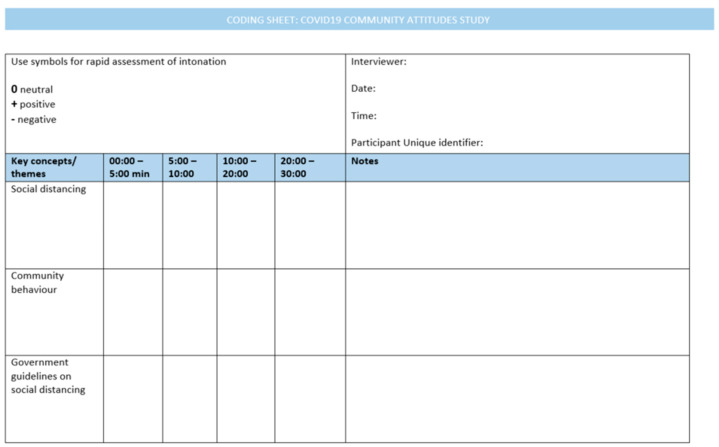
Coding Template (Adapted from Neal et al. [29]).

A coding template was developed in accordance with Neal et al.’s [29] methods and supplied to each investigator. Each investigator was given training in the use of the template. The template included predetermined key concepts and themes related to the research foci, divided the interview into time segments of 5–10 min and provided a legend to assist the interviewer to record intonation and auditory cues. Some investigators wrote reflective notes at the bottom of the coding template. A section of the coding template is provided in Figure 1.

Interview recordings and notes were stored in a shared and secure data repository. The digital notebook provided a feature where the notes could be ‘tagged’ to assist with organisation and classification. These tags were agreed upon prior to interviews commencing, with new tags developed over the course of interviewing as identified by investigators. When new tags were developed, the whole team was notified of their availability. Tags were broad and included terms such as ‘anxiety’, ‘cross-border’, ‘mental illness’, ‘vulnerability’ and ‘cancer’. When each interviewer uploaded their recordings and notes, they were required to tag their notes to create a searchable database that could be used during later analysis.

All interview participants were provided with a unique identifier indicating their interview number_sex_age_MM where MM is their Modified Monash Model location, a recognised Australian measure of rurality rated 1 through 7, with MM1 being a major city and MM7 being very remote [31].

### 2.6. Data Analysis

After all interviews and coding of field notes were completed, a number of key analytical outcomes were agreed upon by the larger collaborative team. These outcomes were developed via collaboration during, and upon completion of, interviews and were broader than the original research foci. Following this, investigators began to work in authorship teams to interrogate more deeply the recordings and notes and to develop greater insight regarding particular ideas.

After an initial discussion of authors TP, LT, ST and AT exploring the theme of healthcare utilization, a set of tags were identified as important to address the aim of this paper. These tags included ‘accessing services’, ‘managing risk’ and specific health concerns including ‘cancer’, ‘anxiety’, ‘chronic illness’, ‘mental illness’, ‘older adult’, ‘pregnancy’ and ‘vulnerability’. An initial trial of the selected tags to verify relevance to the aim and subsequent inclusion was undertaken by one author (TP), by reviewing data that was included and excluded. This was then validated by the review of an additional author (LT) and finally through discussion with authors TP, LT, ST and AT. Based on this process, 59 interviews (out of a possible 90) were chosen for analysis in this paper.

Authors TP, LT, ST and AT were allocated a selection of interviews to review and consider the paper’s foci. Following initial familiarization with the notes, the individual authors considered possible codes and developed themes using a word table template to facilitate data analysis. The completed tables were shared and discussed within the team to confirm the development of themes. Two of the authors (TP and LT) then completed a deeper analysis of the data using agreed themes to ensure the context and meaning of the interpretation was clear. Audio data were reviewed to confirm interpretation as needed. Where further clarification was required, interview data were audio transcribed with transcription software to check meaning and interpretation. All authors reviewed the final in-depth analysis and any discrepancies were discussed within the team until a consensus was reached to confirm meaning. At all stages, transparency of method and discussion were used to promote the validity of findings, rigour and trustworthiness of the process.

### 2.7. Ethics

The original project was approved by the University of Queensland Human Research Ethics Committee (Approval number 2020000800) with reciprocal ethics approval gained from the employing universities of all collaborating researchers.

## 3. Results

Based on the agreed data tags identified during the interview selection process, 59 of the 90 interviews were selected for the data analysis based upon issues related to healthcare access; 31 participants did not raise issues related to access to services. There were participants from all states of Australia and rurality classifications (MM1: 8, MM2: 29, MM3: 3, MM4: 2, MM5: 8, MM6: 3, and MM7: 5). The demographic characteristics of participants are depicted in Table 2.

**Figure 2:**
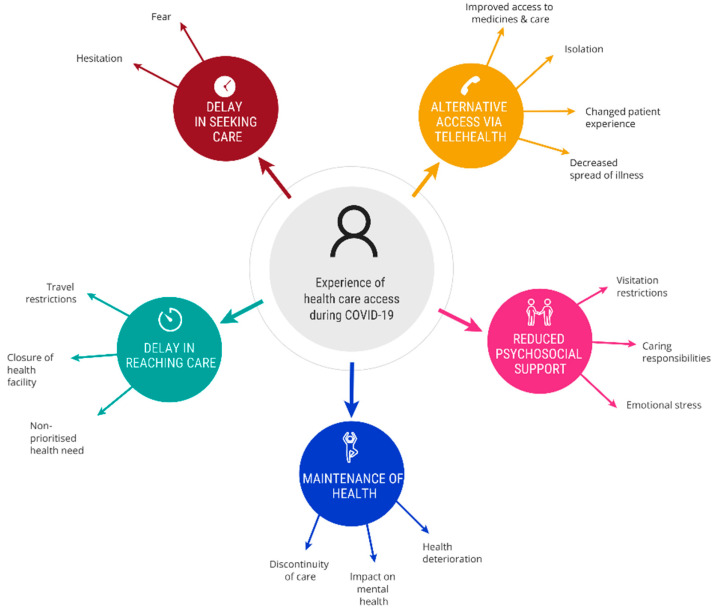
Experience of healthcare access during COVID-19.

Five themes emerged from the interviews (Figure 2): delay in seeking care, delay in reaching care, alternative access via telehealth, reduced psychosocial support and maintenance of health.

### 3.1. Delay in Seeking Care

Participants described many changes to their own as well as family members’ healthcare-seeking behaviours during the COVID-19 pandemic, including avoiding and postponing care. Two sub-themes of choosing to delay care were identified as being related to fear and hesitation.

Fear was mostly connected to the novel virus itself and a belief, even when feeling unwell, that staying home was necessary. In one serious instance, a participant’s mother died from a relatively simple health concern as she had excessive anxiety about going to the doctor:

Mum didn’t die of COVID. She died as a result of the lockdown. She got sick and didn’t go to the doctor because she was scared of going to the doctor because of all the hype on the media about COVID about “don’t go out if you don’t have to”. So she didn’t go to the doctor. It was for a simple urinary tract infection and then she went into renal failure.

(Participant_22_F_52_1)

Some participants delayed seeking care due to hesitation related to generalized uncertainty of the potential changes to travel restrictions and public health messaging, which called for social distancing and limiting outings. For example, one participant recounted living in a region with a GP shortage. She was used to having to wait three weeks to get an appointment and was very conscious of not wanting to take valuable appointment time from others for what she considered were minor health concerns. Hesitation also came about due to the added concern of needing to travel out of regional areas for appointments which required negotiating travel options and leaving an area with no or limited COVID-19 cases.

Some participants felt it was more important to stay at home, rather than seek care for their health condition, although they expressed no issue with fear:

I have the problems you have when you are almost 70, but I have put off going to the doctor because I will do this when this is all over… It’s not such a drama.

(Participant_52_M_68_7)

Delay due to hesitation often reflected this idea of putting up with something that was unpleasant but not life-threatening and seemed to reflect that others needed care more than them, so they should not use resources that others were in greater need of. Sometimes, even when medical care was available locally, it couldn’t be readily solved as illustrated by this woman living in a remote town.

I had really bad allergies. Probably about July, I think it was, it was just super bad. So I had to go down to Perth to get allergy testing. I kind of put it off in terms of I was like, “I’ll get it done in September”… and she [GP] was like “I recommend you go down as soon as possible, because we never know what’s going to happen”.

(Participant_2_F_32_7)

### 3.2. Delay in Reaching Care

Some participants did not voluntarily choose to delay seeking care; rather, they experienced difficulties reaching care. Delayed presentation was due to the impact of interruptions to the health system, including local and interstate travel restrictions and suspension of elective health services. Travel to interstate facilities or bigger urban centres for care presented many challenges. Sometimes what healthcare officials considered ‘not essential’ had a significant impact on those individuals, especially when there was no known date of accessing service. As one participant explained:

So the collagen in my connective tissue is a bit faulty. So for example, I’ve got a torn part of my hip, I’ve had two lots of surgery, and I need another surgical review. I’ve been waiting since May, possibly to get more surgery. But my surgeon, the two surgeons that I can see are in Adelaide, and I can’t go across the border, because it’s not considered like an emergency condition. So instead, I am basically being supplied with painkillers, which isn’t a fun way to live.

(Participant_50_F_37_3)

Other delays in reaching care caused more serious emotional effects, such as this particularly traumatic experience by a 38-year-old mother with an eating disorder:

I needed to go interstate for inpatient psychiatric care, but I wasn’t able to because those facilities had stopped admitting. I wasn’t able to access the care that I really needed. So I did eventually, just last month, get the inpatient admission that I needed, and I am better but there was that big delay of months where the outcome for me would have been much better had I been able to get that admission months ago, but because of COVID, I wasn’t able to. I probably needed it back in February, and I didn’t get it until July. I did end up getting medical admission from April. But what I actually needed was that specialized facility that I couldn’t access. So I was able to basically be prevented from dying, but I didn’t get the psychiatric care that I needed. I feel that it was just terrible timing. And I feel sort of sad for my family, and myself.

(Participant_27_F_38_2)

She went on to report that she had previously generally been able to self-manage her care and stated that *“living in Australia, you always assume that you’re going to be able to get the facilities and services that you need.”* When this was not available, she had ended up with three medical admissions that were *“really hard because the hospital is not really set up to care for that type of patient. It had a really negative impact on me and my family.”*

Delay in access to specialist care was a major issue raised by people who lived away from major metropolitan areas, and the effect was cumulative across multiple types of care, particularly for older people as illustrated by this woman:

We’ve put off going to specialists…. its two and a half hours away from us. Both my husband and I have skin cancers that have to be removed, which we haven’t had done…and optometrists and dental appointments. Yeah. So those sort of appointments have had to be cancelled. Because we can’t travel.

(Participant_7_F_49_3)

### 3.3. Alternative Access via Telehealth

Participants in this study spoke extensively about their experiences accessing telehealth, sharing advantages and disadvantages of this alternative and, in most cases, new method of care. Positive experiences included improved access to medicines and care and avoiding the spread of illness. Telehealth was perceived to improve the reach of health services, allowing for access to healthcare and advice without a need to travel, resulting in an enhanced ability to access specialist care. Many participants spoke about the benefits of accessing a doctor via telehealth for renewal of scripts or advice, seeing this as saving time and the hassle of attending a face-to-face appointment:

…especially when it is just for scripts and stuff like that you know the normal scripts that you get all the time, it’s good to just have that phone consult fax it off to the nearest pharmacy that we go to, it just makes it so much easier, way easier.

(Participant_1_F_34_5)

Another commented:

It’s so much easier for me a full-time working parent…the telehealth appointments have been fantastic.

(Participant_29_F_45_1)

As well as the ease of remote consultations, convenience and the risk of infection was appreciated, as expressed by this woman:

It’s quick, you don’t need to go in and spread germs.

(Participant_59_F_56_5)

Not all experiences were positive. Some participants expressed that it contributed to more isolation and disruption, while others thought that some health services via telehealth were just not as good as in person:

…I myself have had a couple of Drs. over the phone…I don’t think it’s as good, I believe when you go to a Dr. you need to see them and they need to see you.

(Participant_76_F_45_2)

Many participants described conditions or injuries such as a cracked tooth or broken arm that were not suitable for telehealth. For others where a physical assessment was required, telehealth was not possible, which sometimes meant having to access services considered at risk of COVID exposure:

Yeah, they won’t do telehealth because they need to physically examine me. … I had like I had to see a neurologist as well. So that I had to travel to Melbourne to the hotspot because they won’t do telehealth.

(Participant_50_F_37_3)

Even where people were enthusiastic about how doctors had adapted telehealth, many qualified their comments with the need for face-to-face consultations to continue to be available as a choice in dealing with their health concerns. Some participants struggled with accessing healthcare, reporting poorer access to the sort of care they wanted, because they *“couldn’t go to the doctor, all the doctor’s appointments were facetimed or zoom meetings”*. (Participant_55_F_48_5)

Overall, most participants recognized the need for face-to-face consultations for some health issues *“you really do need to have that face to face with the Doctors for a lot of things”* (Participant_1_F_34_5), although not generally elaborating on what problems they felt required face-to-face care.

For those with more complex care needs extending beyond getting test results back or repeat scripts, managing the telehealth consultation required more effort:

My appointments are largely online, we’ve had to plan more than anything. More thought has to go into things. We have to do more assessing…

(Participant_39_F_34_5)

### 3.4. Reduced Psychosocial Support

A critical finding in this study was the reduced psychosocial support available to individuals due to COVID-19 and how this impacted health and wellbeing. Healthcare is often provided or facilitated through family and friends; however, visiting restrictions, caring responsibilities and emotional stresses of supporting others who had serious health conditions were notable. Many touched on the difficulties associated with not being able to visit or receive visitors in care facilities:

It’s really upsetting to see older people really struggling because they can’t have visitors in the nursing homes… I know how difficult it would be to not have anyone come to help or visit.

(Participant_76_F_45_2)

The difficulties mentioned included a lack of support, negative impact on family and relationships and early termination of treatment. For people with caring responsibilities for young children, accessing appropriate care was made difficult due to a lack of support for childcare arrangements. For instance, there were restrictions on being able to take children to appointments and normal support systems were unable to be accessed:

I ended up altogether about six or seven weeks on a medical ward. And because of COVID, my children couldn’t come and visit. My partner could only visit for half an hour a day. And so it was really isolating, as well. I went down to Brisbane to stay in a psychiatric facility for a month, and usually my partner would have flown down and brought the children, but they were only allowing visitors for half an hour on Saturdays and Sundays. And I needed to say there was no point. So I didn’t get to see our children for a month. It was horrible. I mean, it really impacted my feelings on how long to stay for the admission. So I was considering discharging myself after a couple of weeks, even though it really wasn’t long enough. You know, I was worried about how would impact my relationship with my partner and my children.

(Participant_27_F_38_2)

The challenges reported by people related both to the giving and receiving of support. This included concern about asking for in-person assistance for support and the potential risk of COVID-19 transmission. As one mother reported:

I am really stressed and because I can’t ask for the help because I won’t have my parents to watch the kids or babysit.

(Participant_1_F_34_5)

For those with older parents who were used to being able to easily travel to provide check-ups or support, there were additional stresses:

My mum’s been really sick…because of COVID we couldn’t go home to see her, so we didn’t realize how sick she was, that’s been a bit of a problem. She’s here with me now, now that the borders have closed again, she can’t go home…We can take her home but then we can’t come back…

(Participant_76_F_45_2)

### 3.5. Maintenance of Health

Experiences of maintaining health during COVID-19 were varied and there were instances of discontinuity of care, health deterioration and negative impacts on mental wellbeing. Some participants reported feeling that they had to put their health on hold during this time. Feelings of having to “tread water”, “juggle” and manage with sub-optimal access to care and resultant gaps in continuity of care led to both physical and mental discomfort, anxiety and suffering. This was particularly stressful for those with caring responsibilities and those who experienced a significant disruption to a pre-existing routine:

I have my child that has a chronic health condition who was very fearful. The things that he did previously to maintain his health, were no longer available. My son’s health condition did deteriorate not being able to access therapists. And there’s only so much that you can do yourself.

(Participant_31_F_55_5)

So when we first had the lockdown, that was actually my worst time, like, I was crying every day, because I couldn’t swim. And so I was watching my legs just get bigger and bigger. That was probably my most devastating thing… The pool said that they weren’t going to open until September. And that actually tipped me over the edge. I cried I think for three days. I was sore. And, then very fortunately, one of my clients is the principal of a school and she allowed me to swim in the school pool..

(Participant_85_F_54_2)

## 4. Discussion

This study described Australian healthcare utilization experiences during the first eight months of the COVID-19 pandemic. Five themes were developed from the interviews relating to how individuals experienced healthcare access during COVID-19, including delay in seeking care, delay in reaching care, alternative access via telehealth, reduced psychological support and maintenance of health. Within these themes, participants discussed how COVID-19 impacted their ability to access healthcare (both supply and demand) and ensure continuation of care during the pandemic.

The data gathered around postponement of care and the use of telehealth to replace face-to-face appointments during COVID-19 is consistent with other studies, which have demonstrated that these conditions have generally been well accepted [32,33]. Similar to the study by Isautier et al. [32], decisions to cancel or postpone in-person health appointments occurred even when some felt that they actually needed a face-to-face appointment. Regardless of prior health status, delayed access to care can lead to exacerbation of illness and even death. Health can further deteriorate even once patients are able to access healthcare after a delay, and there can be negative longer-term effects for recovery. Furthermore, maintenance of chronic disease generally requires regular access to health services for check-ups and treatments [34,35].

Our findings around delays are mostly in keeping with Thaddeus and Maine’s [36] three-delay model, which includes decision-making around seeking care, reaching a healthcare facility and receiving adequate and timely care. Our study highlights some of the key factors that lead to delays in these areas during a pandemic, including fear and hesitation, travel restrictions, closures of health facilities, non-prioritized health needs, visitation restrictions, caring responsibilities and emotional stress. When delays do occur, this can lead to discontinuity of care and deterioration in a person’s health and wellbeing. Our findings show that alternative ways of delivering healthcare, such as telehealth, can facilitate improved access to medicines and care while ensuring consumers are not at an additional risk of COVID-19. However, such changes need careful consideration as while our results showed they are generally well-received, they change patient experiences of care and can result in increased feelings of isolation.

In line with Levesque et al. [12], these results highlight interactions between supply-side and demand-side determinates of access, and how these interactions can lead to significant health consequences if consumers are unable to easily use services appropriate to their needs. They also show the important role of psychosocial support in the healthcare experiences of participants during the pandemic. This has also previously been highlighted in the access literature. Decisions to seek and engage with healthcare services were in many cases impacted by visiting restrictions and difficulties accessing childcare. In some cases, the psychosocial care normally provided by family and friends was limited, which created additional emotional stressors for the ‘patient’ and their support system. Balancing the need for people to access their normal support system to facilitate access to healthcare against the risk of infection is an important consideration for public health pandemic policies.

These findings are particularly concerning for adults with chronic conditions, especially if they also have a mental health condition, as it has been reported that they are more likely to have delayed or forgone care during the pandemic than those without a pre-existing condition [37]. It is also clear that the pandemic and associated restrictions had serious psychological impacts, including generalized anxiety, worry about infection and becoming unwell and depression [38,39,40]. Such concerns have been reported as potential reasons for not engaging in help-seeking for both mental and physical health issues [41].

It is worth noting that these interviews were completed relatively early in the Australian pandemic, when so much was unclear about the disease and future; it was a time of rapid change and anxiety. The health system was responding based upon the emerging evidence in other countries, where health systems were being overwhelmed by COVID-19 illness, and Australian public health measures were geared toward avoiding such overwhelm. These approaches have shifted over time and with increased understanding of the virus and epidemiology. It might be argued that managing the psychosocial consequences for people has improved with time. For example, following New Zealand’s “bubble” approach [42], social bubbles were introduced and caregiving restrictions were loosened.

### 4.1. Limitations and Future Research

This study used rapid identification of themes from audio recordings (RITA), and while this enabled the research team to analyse a larger number of interviews than is typically included in qualitative research, we acknowledge that this approach has a risk of interpretive bias [43]. Furthermore, due to the changing and evolving nature of the pandemic, people being asked to respond to their experience at one point in time may not capture the complex experience of the entire pandemic on healthcare access over time.

Future longitudinal research to explore people’s experiences of accessing healthcare in Australia throughout the duration of the pandemic would be helpful to understand these issues better. Since this study’s data was collected, case numbers have risen in Australia and some healthcare systems are reportedly becoming overwhelmed.

### 4.2. Recommendations

The development and implementation of health policies and programs during pandemics, or other challenging times where health services may be disrupted, should take into consideration the complex range of factors that impact health seeking and experiences of care. It is important that in preventing pandemic-related health issues and healthcare system overwhelm that new issues are not created that increase individuals’ inability or hesitation to access care. Understanding the way in which messages are understood by different population groups, such as people with underlying conditions that need regular healthcare, requires more sophisticated messaging beyond the avoidance of transmission of the virus. Public health measures are needed to encourage consumers to seek care appropriately during health emergencies, and to support healthcare providers to manage and respond effectively to cases of disrupted access to care.

Telehealth was generally viewed as feasible and acceptable by individuals, supporting further investment and training into this service. Consumer education and support around health technologies as part of pandemic preparedness activities is especially important for those with chronic, pre-existing conditions and for those who live in rural areas who may be affected by border closures. However, telehealth is not a panacea and person-centred care is important for vulnerable persons and groups, where unmet health needs can increase experiences of pain and compromise quality of life.

## 5. Conclusions

This study has described Australian healthcare utilization experiences during the first eight months of the COVID-19 pandemic, highlighting how COVID-19 impacted people’s ability to access healthcare and ensure continuation of care. The findings highlight the complex, interactive and dynamic nature of access during a global pandemic and show how delays in access to healthcare can be influenced by a multitude of factors, which can in turn impact health and wellbeing outcomes. Person-centred approaches that take into account the physical, mental and psychosocial needs of individuals, while balancing the risk of COVID-19 infection are needed to prevent additional health burdens during the pandemic. This includes mechanisms to support people’s decisions to access care in a timely manner during a pandemic, such as alternative methods like telehealth, reassurance of infection safety, exemptions for the need to access elective services and messaging of the importance of continued engagement with health providers.

## Figures and Tables

**Table 1:** Interview Schedule.

**Interview prompts**
Can you tell me about your experiences of life during the COVID-19 pandemic? What are your thoughts about the Australian Government’s social distancing and hygiene guidelines? Can you provide examples of how they affected your everyday life? Do you believe the Australian Government’s social distancing guidelines have been appropriate for your community? Why? What changes have you made/did you make in your everyday life to reduce your risk of infection? Will/have you continue/d these practices after the restrictions are/were lifted? Is there anything you feel you would continue to do in the future after the pandemic has passed? Why? Do you believe there are other things that could be done/could have been done in your community in response to the COVID-19 pandemic? How has everyday life for you, your, friends, family and your community changed as a result of the COVID-19 pandemic? Do you think that positives have emerged from the COVID-19 epidemic? If so, what are they? How has COVID-19 impacted your hopes and dreams for the future?
**Additional probing questions (as needed):**
Could you explain that a little bit more? Could you give an example? Do you think others would feel the same way? How could this be better achieved?

**Table 2:** Demographic characteristics of participants.

	Larger Project	Included in Current Analysis
**Rurality classification**		
Metropolitan (MM1)	12	8
Major regional (MM 2–3)	42	33
Rural (MM 4–5)	17	10
Remote (MM 6–7)	19	8
**Gender**		
Male	20	9
Female	70	50
Prefer not to say	0	0
**Age**		
18–24	3	1
25–34	9	5
35–44	16	11
45–54	23	16
55–64	25	17
65+	13	6
Did not respond	1	1
**State**		
Victoria	7	5
NSW	8	5
Queensland	66	42
ACT	0	0
South Australia	1	1
Western Australia	5	4
Northern Territory	2	1
Tasmania	1	1

## Data Availability

The data is stored in a secure digital repository through The University of Queensland. Approval to access to the repository can be sought through Geoff Argus on g.argus@uq.edu.au.
